# Properties of Slurry Shield Tunnel Sand and Its Application in Large Flow Concrete

**DOI:** 10.3390/ma15155131

**Published:** 2022-07-23

**Authors:** Ba Hezhuoli Ke Zierkailedi, Libo Bian, Xiufang Wang, Xingbo Hu, Xuelei Liu, Zhi Zhang

**Affiliations:** 1 School of Civil and Traffic Engineering, Beijing University of Civil Engineering and Architecture, Beijing 100044, China; 18811317316@163.com (B.H.K.Z.); wangxiufang07@163.com (X.W.); 2 Beijing Shou Fa Tian Ren Ecological Landscape Co., Ltd., Beijing 100000, China; 18811313298@163.com (X.H.); 18518411615@163.com (X.L.); 3 Beijing Shou Fa Expressway Construction Management Co., Ltd., Beijing 100000, China; 17710041226@163.com

**Keywords:** tunnel slag, slurry shield tunnel sand, clay and clay lumps, inert silt, mixed sand, workability, mechanical properties, durability

## Abstract

The amount of Slurry shield tunnel slag (SSTS) from the Beijing East Sixth Ring Road renovation project is about 3 million m^3^, and it is mainly fine and silt sand. In order to realize its resource utilization, the properties of SSTS and the performance of concrete with strength grades from C30-C60, which used the mixed sand compound with SSTS and Coarse Manufactured Sand (CMS) as a fine aggregate, were investigated. The results showed that SSTS’ fineness modulus is 1.2, its clay content is 17.0% but its composition is mainly Inert Silt (IS), and SSTS replaced with 40% of the mass of CMS can obtain a mixed sand with a fineness modulus of 2.7 and a clay content of 7.0%. The morphological and filling effects of SSTS and IS will improve the workability and durability properties of concrete with no adverse effects on the compressive strength. On the other hand, clay lumps in SSTS adversely affect the workability, early cracking properties, and freeze resistance of concrete, which can be alleviated by dewatering and crushing the clay lumps in SSTS.

## 1. Introduction

To meet the rapid development of China’s economy and accelerating urbanization, many underground tunnels have been built in recent years. However, the consequent problem is that a large amount of Tunnel Slag (TS) has become an important part of construction waste [1]. A tunnel 1-km long and 6 m in diameter is estimated to produce about 68,000 m^3^ of TS. For the next 5 to 10 years, China’s subway construction mileage is expected to increase by over 4000 km, which will generate about 2.7 billion m^3^ of TS. Given that the current resource-use rate of TS in China is under 10% [2], the degree and diversity of TS resource-use has great potential for development.

In the early days, TS was mostly disposed of by dumping or landfilling, which can cause the following negative effects [3,4,5]: (a) occupying urban land resources and destroying land surface topography; (b) damaging urban roads during transit and aggravating air pollution; (c) damaging land surface ecosystems and contaminating the soil and groundwater resources; (d) triggering geological disasters, such as landslides and mudslides; (e) blocking surrounding rivers, weakening flood discharge capacity, and affecting the operation of water conservancy facilities; and (f) increasing the economic cost of transporting and occupying the land. To avoid further harm to the ecological environment, TS should be directly disposed of harmlessly and resourcefully after excavation, which can also yield considerable environmental and economic benefits. The efficient resource-use of excavated materials based on soil properties can reduce their disposal costs and carbon emissions by about 85% [6]. This disposal can reduce 14 kg of CO_2_ emissions per ton [7].

TS properties depend on a tunnel’s geohydrological conditions, rock properties, and excavation methods, in which tunnel boring machines and drill-and-blast methods are mostly used in rock formations; their TS contains more solid rock, gravel, and stone chips, which are generally used as concrete aggregate [8,9], inert cement admixture [10], roadwork gravel, roadbed landfill material, agricultural farming material, coating industry raw materials, and filler for various types of land reclamation projects [11,12]. Given that earth pressure balance shield-tunnel construction will require additives, such as surfactants, polymers, bentonite slurry, anti-wear agents, and defoamers, the effective removal of such additives in the excavated tunnel slag is the key point of its resource-use [13]. Moreover, treated TS can be used for concrete aggregate, embankment material, land backfill material, and synchronous grouting material [14].

In the slurry shield tunnel construction, the slurry recycling system consists of slurry preparation, slurry adjustment, slurry transfer, and slurry–water separation [15]. Firstly, the slurry prepared by water, clay, bentonite, and flocculant forms a mud film to seal the working surface and balance the soil pressure [16]. Thereafter, slag-carrying slurry is pumped into the ground, and the treatment process mainly includes pre-screening, cyclone screening, flocculation and sedimentation, centrifugal separation, vibration dewatering, and press filter dewatering, which can discharge TS in the form of stone, sand, mud, and mud cake. Given the high content of fine sand, silt, silty clay, and clay in most slurry shield TS, it is often used to prepare shield slurry, post-wall synchronous grouting materials [17], sintered bricks, no-burn bricks, ceramic granules, and planting soil [18].

Given the global shortage of natural sand and gravel resources, materials other than natural aggregate should be sought, but research on the resource-use of slurry shield TS in the concrete aggregate field is insufficient. Some studies have shown that the high-value sand and stone separated from slurry shield TS can be used as concrete aggregate [19]. However, what must be ensured is that the bentonite content in sand with a particle size >30 μm should not exceed 1% [20]. With the current maturity of the slurry–water separation equipment and process, the performance of slurry shield tunnel sand (SSTS) obtained after separation tends to be stable. Using geological surveys and geotechnical analysis to establish a model to predict the yield and type of TS that will provide effective guidance for the construction plan and equipment selection and adjusting the equipment in real time after the sampling results of the in situ TS, a harmless TS can be obtained that can be easily sorted, stored, and reused [21]. Although SSTS particles are fine, research on the preparation of concrete using desert sand [22], oil sand waste [23], and special fine sand from waterway remediation [24] has indicated the feasibility of preparing concrete by partially replacing fine aggregates with SSTS.

The use of SSTS as an alternative material to concrete fine aggregate, which can reduce the negative environmental impact of TS and the wastage of sand resources, will provide considerable economic, social, and environmental benefits to tunnel projects. This paper tested the physical and chemical properties of SSTS; evaluated the effects of SSTS partially replacing the mechanism sand with respect to the workability, mechanical properties, and durability of large flow concrete; and explored the feasibility of preparing large flow concrete by SSTS.

## 2. Materials and Methods

### 2.1. Materials

#### 2.1.1. Cementitious Materials

Cementitious materials used in this paper consisted of cement, fly ash, and blast furnace slag with mass ratio of 6:2:2. These materials are described as follows: P.O 42.5 ordinary Portland cement with standard consistency water consumption of 27.8%, 80-μm sieve residual of 2.8%, 28-d compressive strength of 44.5 MPa, and 28-d flexural strength of 7.1 MPa; class II fly ash with 45-μm sieve residual of 13.2%, 5.65% burn-off, and 102% water demand ratio; and S95 grade blast furnace slag with 45-μm sieve residual of 4.00% and 91.9% flowability.

#### 2.1.2. Aggregates

The tunnel of the East Sixth Ring Road renovation project is located in the lower part of the alluvial fan of Yongding River and Chaobai River in Tongzhou District. The stratum along the route is mainly composed of sand layer, fine-powder sand layer, silt layer, silt clay layer, and round-gravel pebble layer interlayer deposits. Geological exploration results showed that TS contains about 40% silica–alumina sand particles.

**Figure 1:**
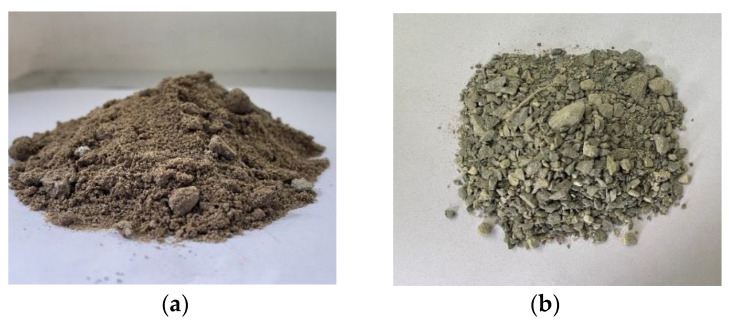
Appearance of sand used in this study: (**a**) SSTS; (**b**) M-S.

**Figure 2:**
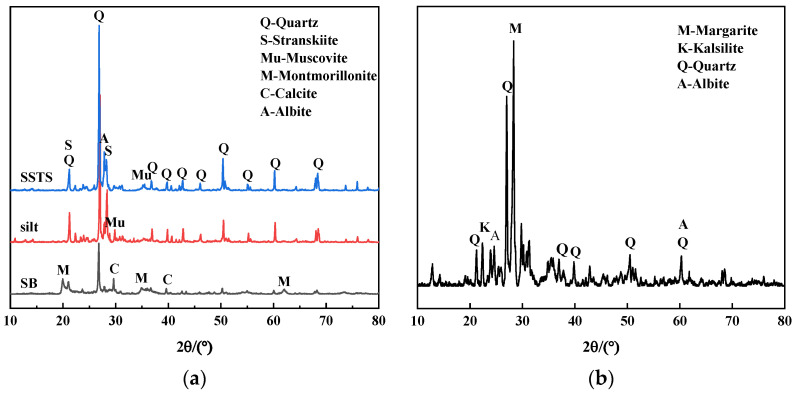
XRD pattern of materials: (**a**) SSTS, silt, and SB; (**b**) M-S.

**Figure 3:**
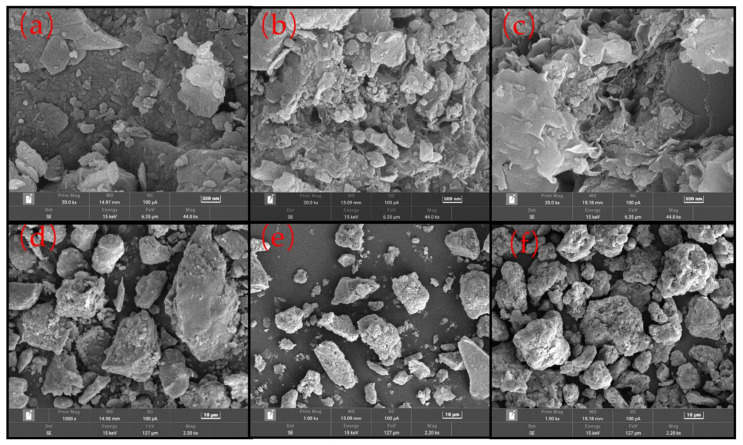
SEM images of materials: (**a**) silt with particle size under 75 μm in SSTS (500 nm), (**b**) stone powder in M-S (500 nm), (**c**) sodium bentonite (500 nm), (**d**) silt with particle size under 75 μm in SSTS (10 μm), (**e**) stone powder in M-S (10 μm), and (**f**) sodium bentonite (10 μm).

**Figure 4:**
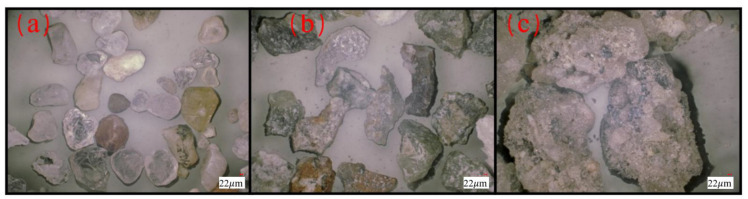
Ultra-deep field body microscopy images of materials: (**a**) washed SSTS with particle size of 0.3–0.6 mm, (**b**) washed M-S with particle size in 0.3–0.6 mm, and (**c**) unwashed SSTS with particle over 1.18 mm.

This study used two types of sand as raw materials for concrete fine aggregate, as shown in Figure 1. The first type of sand is SSTS obtained from the tunnel site. Its physical properties—which include fineness modulus, specific granularity, apparent density, loose bulk density, dense bulk density, loose porosity, dense porosity, clay content, clay lump content, chloride ion content, organic content, and water content—were tested using Chinese national standard GB/T14684-2011 [25]. The results are shown in Table 1. As shown in Table 2 and Figure 2, the chemical and mineral compositions of three materials were tested by XRF and XRD, respectively, including SSTS, and silt with particle size below 75 μm in SSTS and Sodium Bentonite (SB), which is a raw material in shield slurry preparation. The microscopic characteristics observed by SEM of the silt, stone powder in Manufactured sand (M-S), and SB are shown in Figure 3. Figure 4 shows the morphological characteristics of the particles of washed and unwashed SSTS, and the washed M-S observed via ultra-deep field body microscopy.

The second type of sand is granite M-S produced in Beijing, which has a fineness modulus of 2.6, loose porosity of 44%, and methylene blue value of 3.5. Mineral compositions of M-S are shown in Figure 3b and particle morphological characteristics of M-S are shown in Figure 4.

**Figure 5:**
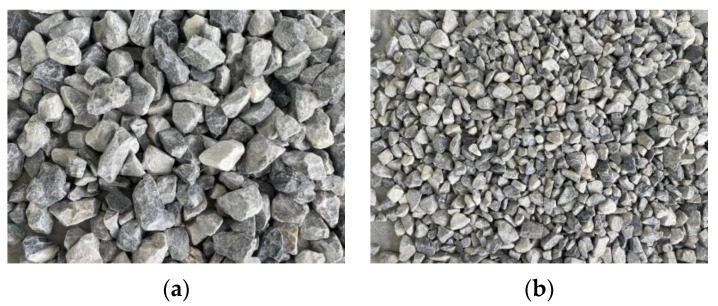
Appearance of the used coarse aggregate: (**a**) particle size in 10–25 mm; (**b**) particle size in 5–10 mm.

In this paper, crushed stone with two particle sizes of 5–10 mm and 10–25 mm were formulated as concrete coarse aggregate according to a mass ratio of 4:6, as shown in Figure 5. Aggregate with particle size of 5–10 mm has apparent density 2670 kg/m^3^, clay content 0.3%, and crushing value 5.6; aggregate with particle size of 10–25 mm has apparent density 2690 kg/m^3^, clay content 0.4%, and crushing value 5.4.

Table 1 shows that the overly fine particles and excessive content of clay and clay lumps are the main factors limiting SSTS’ application in concrete. In the requirements of GB/T14684-2011, particles under 75 μm in size are defined as clay powder, and the clay mineral particles in them are considered to have adverse effects on concrete performance. As shown in Figure 2, the XRD patterns of SSTS and silt are basically the same, its main mineral compositions are Quartz, Stranskiite, Albite, and Muscovite; nevertheless, SB is composed mainly of Montmorillonite. In addition, as evident in Table 2, there are no distinct toxic heavy metal elements in SSTS, the silt, and SB. As shown in Figure 3a–c, SB particles’ surface have obviously laminar convoluted structure and microscopic pores, and the stone powder particles have a rougher surface than the silt particles. At the same time, we can see in Figure 3d–f that the particle morphology of the silt and stone powder is significantly different from that of SB. Considering the test results, we tentatively consider that particles below 75 μm in SSTS are inert silt (IS). This study used SSTS to replace coarse mechanism sand (CMS) and investigated the synergistic effect of the two fine aggregates on various properties of concrete.

#### 2.1.3. Water Reducer and Mixing Water

The water reducer used in this research process is polycarboxylic acid water reducer provided by Beijing Shou Fa Tian Ren Ecological Landscape Co., Ltd. (Bejing, China), with water reduction rate of 28%, and tap water from the materials laboratory of Beijing University of Civil Engineering and Architecture was used as mixing water.

### 2.2. Mix Proportions

**Figure 6:**
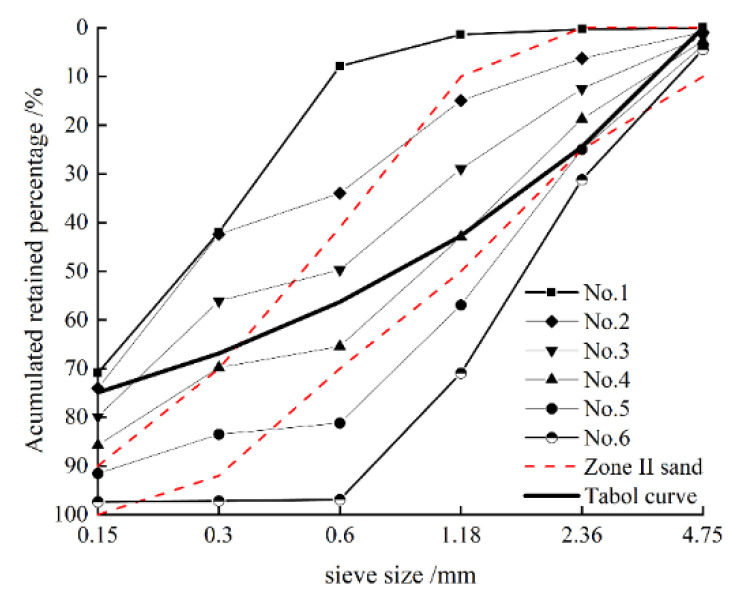
Grading curve of each mixed sand. Range of sand in zone 2 in the figure is the gradation range of mechanism sand in GB/T14684-2011. Table curve in the figure is based on the following formula: $L_{i}=1-P_{i}=100\times {\left(\frac{d_{i}}{D}\right)}^{n}$, where $L_{i}$ is the accumulated sieve rate, $P_{i}$ is the passing rate, $d_{i}$ is the diameter of each sieve, *D* is the maximum particle size, and *n* is the experimental index.

Since the particle size of SSTS is mostly less than 0.60 mm, its compounding with M-S will lead to a poor gradation and to an excessive amount of powder. Hence, fine particles under 0.60 mm in M-S were screened out, and CMS with fineness modulus of 3.8 was obtained. Thereafter, CMS was replaced by SSTS according to mass ratios of 0, 20, 40, 60, 80, and 100%. Properties of each type of mixed sand are shown in Table 3. The grading curve of each type of mixed sand is shown in Figure 6. Fineness modulus and grading of the No. 3 mixed sand are similar to M-S, and they fit better with the Table’s grading curve at *n* = 0.4. Nevertheless, clay content of No. 3 mixed sand is 7%, which exceeds the maximum limit of 5% in GB/T14684-2011. However, properties of IS and clay mineral are relatively different. Hence, the mass ratio of 4:6 was used as the optimal ratio of SSTS to CMS compounding.

As shown in Table 4, dry mixed sand (DMS), wet mixed sand (WMS), and M-S were used as fine aggregates in the concrete preparation test, and four concrete proportions of strength classes C30, C40, C50, and C60 were designed according to JGJ 55-2011 [26]. In each strength grade, only the type of fine aggregate has been modified, and the proportion of the rest of the materials remained the same. Meanwhile, the proportion of WMS groups were adjusted to the moisture content of wet SSTS on the test day, and the mix proportions are shown in Table 5.

### 2.3. Test Methods

Chemical compositions of SSTS, IS, and SB were tested by X-ray fluorescence (XRF) (Supermini model manufactured in Japan, elemental detection range from F to U); mineral compositions of SSTS, IS, M-S, and SB were tested by X-ray diffractometer (XRD) (Ultima VI model manufactured by RIKEN, Tokyo, Japan) with scanning speed of 5°/min and test range of 10–80°. Microscopic morphology of IS, M-S powder, and SB were characterized by scanning electron microscope (SEM) (TESCAN MIRA LMS, Brno, Czech Republic), and the particle morphology of SSTS and M-S were observed by ultra-deep field body microscopy (Keenes VHX-2000 model, Osaka, Japan) with magnification of 0.1× to 5000×.

Workability was evaluated by the slump and expansion values according to the requirements of the Chinese national standard GB/T50080-2016 [27]. Specimens with sizes of 100 mm × 100 mm × 100 mm and 100 mm × 100 mm × 400 mm were used to test the cubic compressive strength (at ages 3, 7, 28, and 60 d) and flexural strength (at age 28 d), respectively. Three specimens were prepared for each group, and the average value was taken as test result; the testing process was in accordance with the requirements of GB/T50081-2019 [28]. Those tests were carried out at a conditional temperature of 20 ± 5 °C and relative humidity exceed 50%.

Concrete durability was tested according to the requirements of the Chinese national standard GB/T50082-2009 [29], including recording contact shrinkage performance, curing at standard conditions (temperature of 20 ± 2 °C; relative humidity exceeding 95%) for 3 days, and by recording shrinkage values at 3, 7, 14, 28, 45, and 60 d in a room with a constant temperature and humidity (temperature of 20 ± 2 °C; relative humidity of 60 ± 5 °C), with each group of three specimens having a size of 100 mm × 100 mm × 515 mm.

For testing the anti-chlorine ion erosion performance, after 28 days of curing in standard conditions, vacuum water saturating for 24 h, and then using the coulomb electric flux method, each group of three specimens with the size of 50 mm × 100 mm × 100 mm was evaluated using NEL-PDU type chloride ion diffusion coefficient tester.

To test the water penetration resistance of concrete, six specimens were prepared for each group, and their penetration depth under water pressure after a 28-d curing at standard conditions was measured.

The carbonation resistance was tested, after 26 days of curing in standard conditions and 48 h drying at 60 °C in an oven, via carbonizing each group of nine 100 mm × 100 mm × 100 mm cubic specimens at 3, 7, and 28 d in the carbonization chamber (CO_2_ concentration of 20 ± 3%, relative humidity of 70 ± 5%).

To test the freeze resistance, after 24 days of curing in standard conditions and 96 h soaking, a rapid freeze–thaw test was conducted by recording every 50 times freeze–thaw cycle to test the transverse fundamental frequency and quality of the test specimens, one freeze–thaw cycle takes 2–4 h. Each group of three specimens had a size of 100 mm × 100 mm × 400 mm.

By using the circular cracking test, the first cracking time and width of the first cracks of concrete under the action of circular binding force and early shrinkage stress were determined. In addition, this test was conducted after curing at in outdoor conditions with average temperature of 28.5 °C and relative humidity of 81%.

**Figure 7:**
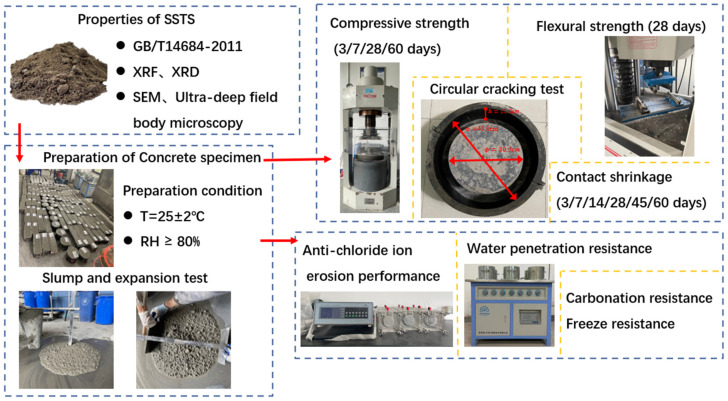
Main methodology flow chart.

The main methology flow and the appearance of test equipments are shown in Figure 7.

## 3. Results

### 3.1. Workability

**Figure 8:**
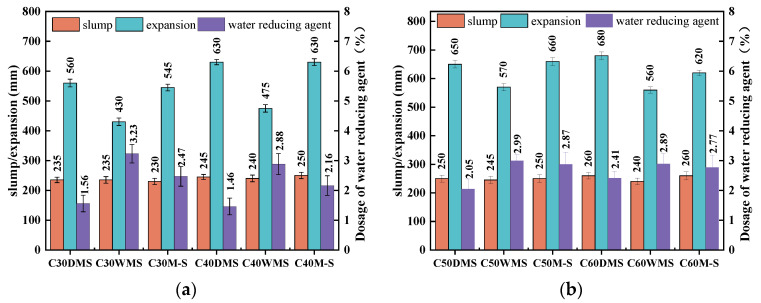
Workability of concrete mixtures of each group. (**a**) Workability of C30 and C40 strength grade. (**b**) Workability of C50 and C60 strength grades.

Figure 8 shows the value of the slump, expansion tests, and water reducer dosing of each group. The slump and expansion values of the concrete mixture were maintained at 230–260 mm and 500–650 mm, respectively, by adjusting the water reducer dosing, ensuring that the slump of each group was within 230–260 mm, and then recording the value of its expansion. In each strength grade, DMS groups show the best fluidity with the least amount of water reducer dosing, the value of the expansion of the WMS groups is the lowest with the largest water reducer dosing, and the M-S groups have a median water reducer dosing between the WMS and DMS groups and its fluidity values are the same as those of DMS groups.

**Figure 9:**
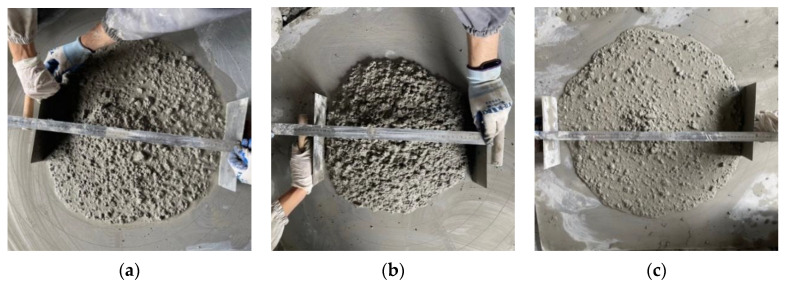
Appearance of concrete mixture of C30 strength grade. (**a**) C30DMS. (**b**) C30WMS. (**c**) C30M-S.

Figure 9 shows the appearance of concrete mixture of a C30 strength grade; C30DMS displays better workability, other strength grades show the same regularity.

The water reducer dosings in the M-S groups in C30–C60 concrete were 158%, 148%, 140%, and 115% higher, respectively, than those of the DMS groups. This result mainly comes from two aspects: SSTS particles have more regular morphological characteristics, mostly round or oval, while M-S particles have irregular morphological characteristics and poor angularity, as shown in Figure 4. Therefore, SSTS can easily slip into the voids in the aggregate [30], which is proven by the fact that the loose porosity of DMS is 4% lower than that of M-S. Thus, the DMS groups only need minimal cement paste to fill in the aggregate voids, and those results can increase the surplus of cement paste to promote the workability of the concrete admixture.

The IS’s surface is smoother and its particle shape is more rounded compared with the M-S powder, as shown in Figure 3. Hence, IS can act as lubrication and a micro-rollerball, further filling in the tiny voids inside the concrete, releasing more free layer water [31,32], reducing the friction between the cement paste and the fine aggregate and the mortar and the coarse aggregate, and lowering the viscosity and yield stress of the mortar, thereby improving the workability of concrete admixture [33]. Moreover, the active effect of granite-based mechanism sand powder will significantly increase slurry viscosity and water reducer dosing.

The shield process adds ecologically harmless Polyacrylamide (PAM) as a flocculant, which can play a selective flocculation role in the cyclone process [34], particularly separating inert microfine sand particles from clay particles that should be recovered for reuse. This process will result in minute amounts of PAM remaining in the SSTS, thereby increasing the natural dewatering time of SSTS and forming partial sand clusters with weak bonds and clay clumps that are difficult to disperse (see Figure 4c). The fine particles in the DMS are uniformly distributed and its clay lump content is considerably low. Meanwhile, it is evident that the clay lumps in SSTS exhibit distinct porous characteristics in Figure 4c; we speculate that due to their water-absorbing effect, the clay lumps will absorb part of the water reducer molecules in the mixing process, and this may be one of the reasons for the larger amount of water reducer in the WMS group.

### 3.2. Mechanical Properties

**Figure 10:**
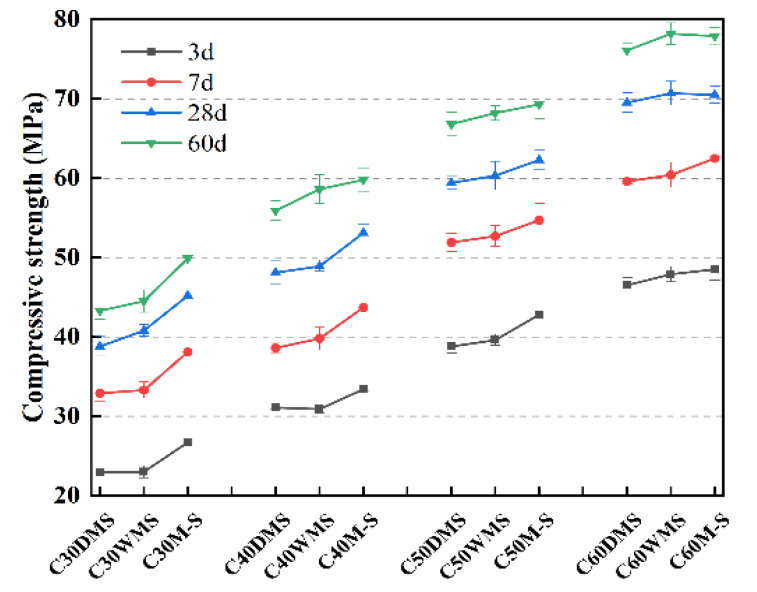
Comparison of compressive strengths of concrete in each group at different ages.

The compressive and flexural strengths of the concrete are shown in Table 6. Figure 10 shows that in each strength grade, the compressive strength at all ages of the DMS and WMS groups is generally similar and lower than that in the M-S groups. In addition, *t*-statistical tests (calculated by origin 2021b) were used to evaluate the difference in compressive strength of each group at 28 d. The test’s null hypothesis assumed that the compressive strength of each group is equal, with a two-tailed significance level of 0.05. The null hypothesis should be rejected if $\left|t\right|\geq t_{0.975}\left(DF\right)$, which means there is a significant difference between the two groups. Meanwhile, in the t-statistical test, the degree of freedom (DF) depends on the standard deviation, so different DFs could be observed in each group with the same sample number.

Table 7 shows no significant difference in the compressive strength of each group in the C50 and C60 strength grades. In addition, the compressive strengths of the DMS and WMS groups in the C30 and C40 grades are significantly lower than those of the M-S groups in the *t*-statistical test.

Note that with an increase in the strength grade, the proportions of fine aggregate particles with a particle size below 0.60 mm to the total mass of the concrete were 14.2, 12.6, 10.9, and 10.8%. Therefore, the degree of influence on the compressive strength of concrete was decreasing. That is, SSTS has an evidently adverse effect on the compressive strength of concrete when the mass ratio of SSTS to concrete mix exceeds 12.6%.

According to JGJ55-2011, the 28-d designed compressive strengths of each strength grade were 38.2, 48.2, 59.9, and 69.9 MPa. Except for some DMS groups, for which the 28-d compressive strengths were slightly lower than the designed strengths, the remaining groups of concrete with cement accounted for only 60% of the mass ratio of the cementitious material required to meet the design requirements.

The crystalline nucleation and activity effects of the M-S powder can play the role of a partially active admixture and increase the generation of hydration products to improve the compressive strength of concrete. Meanwhile, the silt composed of SiO_2_, which is crystalline inert, is essentially still a siliceous sand as natural sand, so there is basically no chemical reaction between IS and cementitious materials [35]. However, the good particle shape of the SSTS sand particles in DMS and WMS can optimize the aggregate gradation of concrete and reduce porosity. Filling, micro-rolling beads, and heterogeneous nucleation effects of IS can improve the particle size distribution of the cementitious material system, thereby optimizing the spatial structure of the cementitious system of the hydration products [36,37]. Meanwhile, those effects of IS can release filling water to further promote hydration, so that the compressive strengths of DMS and WMS can reach the design requirements in the absence of the active effect of M-S powder [38,39].

**Figure 11:**
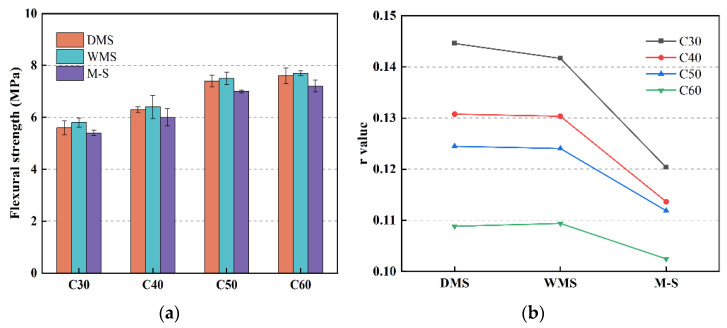
Flexural strength and r value of each group: (**a**) 28-d flexural strength; (**b**) comparison of the r value in different fine aggregates.

Figure 11 shows that the flexural strength and r value of the DMS and WMS groups displayed little difference, but those of the M-S groups are significantly lower. The brittleness of concrete is a form of energy consumption; in the process of energy transformation, the maximum elastic energy accumulated within the material before the fracture critical point is rapidly converted into the main crack fracture as surface energy, and the toughness of concrete is an opposite index to its brittleness [40]. As we know, the r value is the ratio of flexural strength to compressive strength of concrete; the higher the r value, the greater the toughness of the concrete. This result indicates that the flexural strength and toughness of concrete can be improved by using the SSTS compound with CMS. The reason is the lower void ratio of DMS and WMS, which improves the compactness of concrete. Meanwhile, the presence of a moderate amount of IS can further improve the microfine structure inside concrete and enhance the performance of the interfacial transition zone [38]. The presence of a small amount of PAM also enhances the flexural strength, toughness, and interfacial transition zone of concrete [41]. Furthermore, the higher compressive strength of the M-S groups is one of the reasons for its lower toughness.

### 3.3. Shrinkage and Circular Cracking Properties

**Figure 12:**
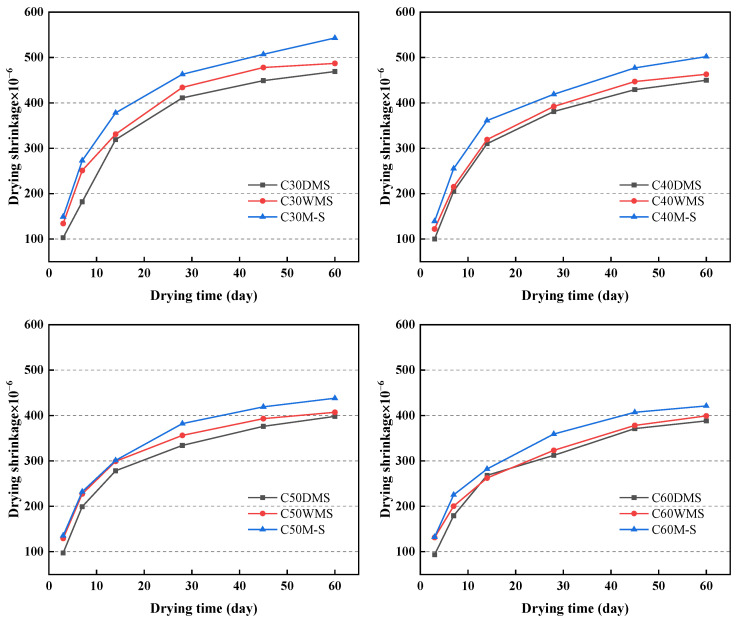
Drying shrinkage of concrete in each strength grade.

This study tested the 60-d drying shrinkage of concrete in a shrinkage chamber after curing for 3 d under standard curing conditions. Figure 12 presents that among the strength grades, M-S groups showed the highest drying shrinkage in the range of 534 × 10^−6^ − 421 × 10^−6^, and WMS groups showed medium drying shrinkage between M-S and DMS groups in the range of 487 × 10^−6^ − 399 × 10^−6^. However, the lowest drying shrinkages were shown in the DMS groups in the range of 469 × 10^−6^ − 388 × 10^−6^. Note that with an increase in the water–cement ratio, the drying shrinkage of concrete and the influence of fine aggregates on the drying shrinkage decreases.

M-S with particle size of 0.60–4.75 mm and gravel with particle size of 4.75–25.00 mm play important roles in inhibiting the shrinkage of concrete [42]. Sand particles under 0.60 mm have a limited size effect and small mass ratio in concrete, thereby leading to their minimal effect on shrinkage [43].

However, the combination of SSTS and CMS improved the particle size distribution and filling compactness of the solid particles inside the concrete [44]. Moreover, the bulk densities of DMS and WMS were less than that of M-S, thereby occupying more volume with the same mass of admixture and increasing the contact points of solid particles, enhancing the skeletal role of aggregates, and improving the volume stability of the concrete. IS can also allow cement particles to be uniformly dispersed and optimize the spatial network structure of the cementitious material, thereby improving the internal pore structure of concrete and reducing its drying shrinkage [45]. We also speculate that the presence of IS can reduce the number of harmful pores inside the concrete, thus achieving the inhibition of concrete shrinkage [46].

The clay lumps in WMS have a porous structure, and water in their internal pores will gradually evaporate, thereby producing large shrinkage deformation. However, the clay-lump content in total concrete is extremely low. Therefore, the drying shrinkage of the WMS groups is higher than that of the DMS groups, but still less than that of the M-S groups.

Table 8 shows that in each strength grade, the first cracking time of the WMS groups is the shortest and its initial crack width is the largest, followed by the M-S groups. The first cracking time of the DMS group is the longest and its initial crack width is the smallest. Meanwhile, the trend of all concrete groups show that cracking time decreases and crack width increases with an increase in water–cement ratio.

The results of the circular cracking test reflect the early cracking performance of the concrete. SSTS and IS with no activity effect in the DMS groups can increase the aggregate compactness and volume stability of concrete, thereby leading to less chemical and plastic shrinkage and better early cracking resistance. The active effect of the M-S powder and the large void ratio of M-S increase the chemical and plastic shrinkage of concrete at an early age; thus, there is an earlier cracking time of the concrete. The presence of a clay lump in WMS is an unstable cracking-inducing factor within the concrete. Under circumferential restraint and shrinkage stress, initial cracks in the clay lump and weak surrounding initial cracks in the concrete will develop rapidly, leading to an earlier cracking time. This result indicates that clay lumps in WMS have a substantial adverse effect on the early cracking performance of concrete.

### 3.4. Durability

#### 3.4.1. Anti-Chloride Ion Erosion Performance and Water Penetration Resistance

**Figure 13:**
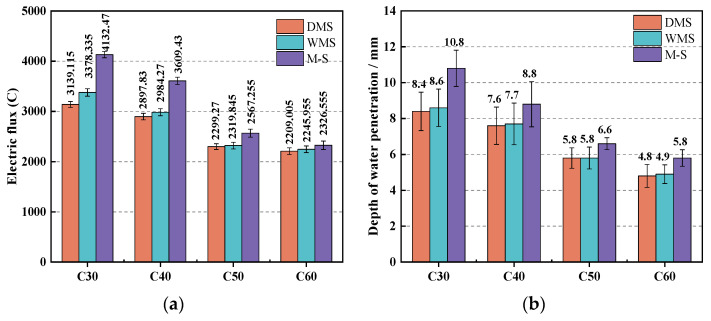
Anti-chloride ion erosion performance and water penetration resistance of each group: (**a**) electric flux of concrete; (**b**) depth of water penetration of concrete.

Figure 13a shows that in each strength grade, the electric fluxes of the DMS and WMS groups are similar, but both are significantly smaller than those of the M-S groups. This result indicates that the use of DMS and WMS can significantly improve the chloride ion penetration resistance of concrete. Owing to the fine particles and rounded shape of SSTS, which can simultaneously reduce the void ratio of the aggregate system and improve compactness, building on these characteristics, we deduced that the filling and lubricating effects of IS can improve the pore structure inside the concrete and reduce the number of contiguous pores. Thus, the chloride ion erosion resistance of concrete is further improved.

The water-penetration resistance of concrete can also indicate its penetration resistance and denseness. Figure 13b shows that in each strength grade, the depths of water penetration of the DMS and WMS groups were similar, in which both are significantly lower than that of the M-S group. These results are similar to the electric flux test. The electric flux test used the central part of the concrete specimen after cutting and further shows the internal integrity of concrete towards resisting penetration. By contrast, a water penetration test measures the effect of the surface layer of concrete to resist water pressure penetration. The results of the two tests show the same outcomes, further demonstrating the optimization effect of SSTS and IS on the denseness, internal pore structure, and penetration resistance of concrete.

#### 3.4.2. Carbonation Resistance

**Figure 14:**
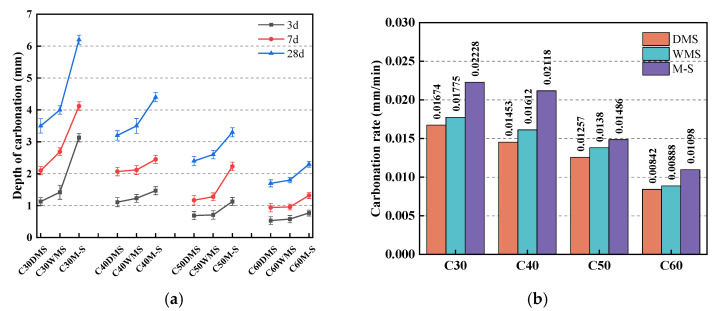
Carbonation resistance of each concrete group: (**a**) depth of carbonation; (**b**) carbonation rate.

The carbonation depths of the concrete at 3, 7, and 28 d are shown in Table 9. Figure 14a shows that among the strength grades, M-S groups showed the largest carbonation depth in each age, and that the DMS and WMS groups showed smaller carbonation depths than the M-S groups. Meanwhile, the smallest carbonation depths were shown in the DMS groups. By evaluation via empirical relational regression analysis, the relationship between the carbonation depth and time can be presented [47] as follows:

(1) $$d=k\times \sqrt{t},$$

where *d* is the depth of carbonation (mm), *k* is the carbonation rate constant, and *t* is the time of carbonation (min). The depth of carbonation at each age is the vertical coordinate and the square root of the carbonation time is the horizontal coordinate. Taking a linear fit to the carbonization data, the slope of the fitting result is the value of *k*.

Figure 14b shows that at each strength grade, the carbonation rates of the M-S groups are the largest, and those of the DMS and WMS groups are significantly lower. In a carbonation chamber with a high CO_2_ concentration, the carbonation products of calcium carbonate and calcium silicate are generated because of the reaction of CO_2_ with calcium ions and the decomposition of calcium–silicate–hydrate, respectively [48]. Additionally, the microstructure of the specimens can become denser after carbonation [49], and this will cause a gradual decrease in the rate of carbonation. In this test, with the same CO_2_ concentration and temperature, given the filling and morphological effect of SSTS and IS in DMS and WMS, we speculated that the smaller void ratio of DMS and WMS will optimize the porosity and permeability of concrete, and reduce the number of fine pores and capillaries inside concrete [50]. Consequently, DMS and WMS groups showed a significantly lower depth of carbonation and carbonation rate. In relative terms, the larger void ratio of the M-S concrete aggregates and the poorer internal pore structure of concrete render it less resistant to carbonation.

#### 3.4.3. Freeze Resistance

**Figure 15:**
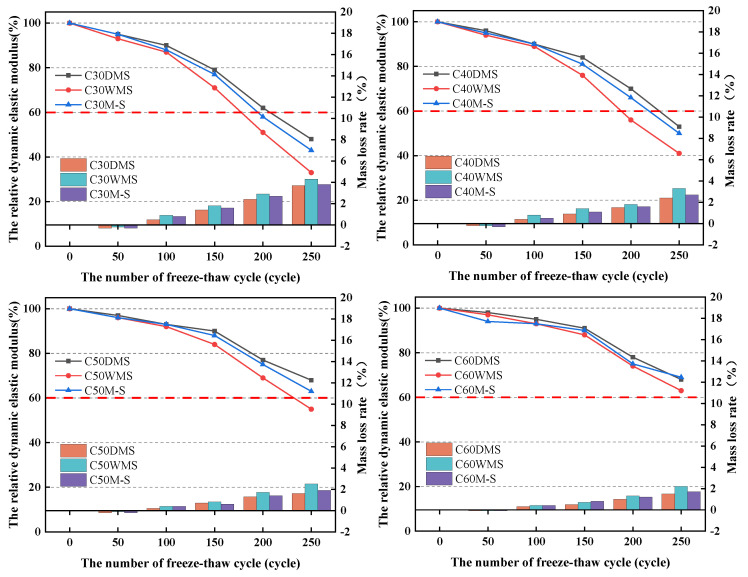
Relative dynamic elastic modulus and mass loss in each group. According to the requirements of GB/T50082-2009, freeze thaw cycle test stop when the relative dynamic elastic modulus of specimen lower than 60%, which is lower than the rad dotted line in the figure.

Figure 15 shows that the freeze resistance of concrete increases gradually with an increase in the strength grade. At each strength grade, the loss of the relative dynamic elastic modulus and mass were the smallest for the DMS groups, and their resistance classes to freeze–thaw could reach F200, F200, F250, and F250. The freeze resistances of the M-S groups were better than those of the DMS groups but less than the WMS groups. Moreover, their resistance class to freeze–thaw could reach F150, F200, F250, and F250. The WMS groups showed the worst freeze resistance, and their resistance class to freeze–thaw could reach F150, F150, F200, and F250.

**Figure 16:**
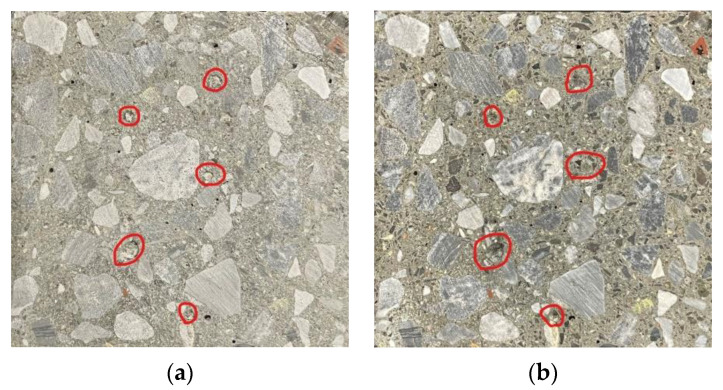
Cross-section appearance of specimen of C30WMS. (**a**) unwashed, (**b**) washed.

During the freeze–thaw cycle, the combined water in the internal pores of concrete undergo a periodic phase change, and the expansion stress will continuously expand the original pores, resulting in new cracks and pores. As the freeze–thaw cycles are carried out, old and new cracks and pores will be further developed, causing the formation of large cracks through the concrete due to freeze expansion and surface peeling [51]. During the test, a higher dynamic elastic modulus and mass loss indicate more damage fractures within the concrete [52]. Clay lumps are porous structures with a significantly lower strength than cement stone and aggregates. As shown in Figure 16, there are obvious clay lumps present in the cross-section of the C30WMS specimen and given its weak porous structure it can be easily washed. Meanwhile, clay lumps will become an unstable point inside the concrete. These lumps will be immediately destroyed when expansion stress is generated in the periodic phase changes, causing the formation of larger defective points inside the concrete, and leading to a rapid development of initial cracks around them.

**Figure 17:**
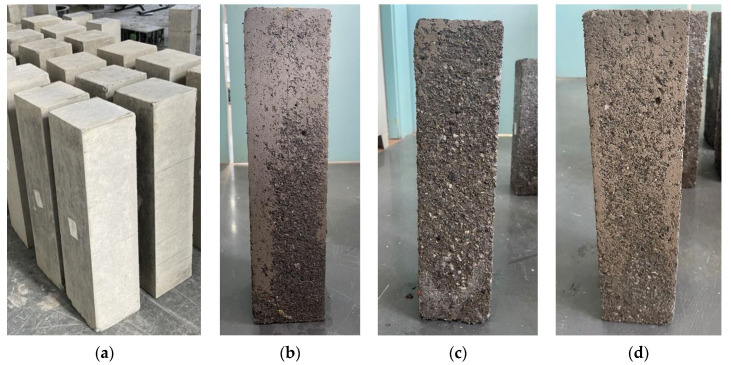
Appearance of concrete specimen after 250 freeze–thaw cycles: (**a**) Pre-test specimens, (**b**) C30DMS, (**c**) C30WMS, and (**d**) C30M-S.

As shown in Figure 17, we have compared the surface of three concrete specimens of C30 strength grade after 250 freeze–thaw cycles; thus, it is obvious that the surface of C30WMS specimen suffers the most severe damage, which indicates that the clay lumps in WMS have a significant adverse effect on the freeze resistance of concrete.

## 4. Discussion

The Chinese national standard GB/T14684-2011 considers particles with a particle size under 75 μm as clay powder, which will have a large adverse effect on concrete performance owing to its special laminar structure. Meanwhile, IS with a particle size below 75 μm in SSTS has a limited adverse effect on concrete and even has a positive effect when mixed in the appropriate amounts.

The concrete test results showed that treatment of SSTS with dewatering, the crushing of clay lumps, and compounding with CMS can solve the problems of significantly fine SSTS particles and high clay and clay lump contents. Moreover, it can be used to prepare large fluid concrete with good performance. Although the direct use of wet SSTS compounded with CMS can adversely affect workability, early cracking performance, and the freeze resistance of concrete, it can only be used in concrete element plants and project sites for the preparation of small concrete members and non-load-bearing parts of concrete. Retreatment at the tunnel site to further reduce clay content and clay lump content of SSTS can further increase its resource potential.

## 5. Conclusions

This paper studied the physical and chemical properties of SSTS, and the performance of concrete using mixed sand compounding with SSTS and CMS as fine aggregate. The following findings can be drawn according to the previously discussed test results.

- An ultra-fine particle size and a high clay content are the main characteristics of SSTS. In SSTS, the chemical composition of IS with a particle size under 75 µm is similar to sand particles with a particle size above 75 µm. In addition, its mineral composition is mainly quartz, feldspar, and muscovite. Microscopic test results show that SSTS and IS have better morphological characteristics and microscopic morphology compared with M-S. The best results of compounding SSTS with CMS at a mass ratio of 4:6 can obtain mixed sand with a fineness of 2.7 and a loose porosity of 40%.
- By adjusting the water reducer dosage, the slump values of C30–C60 strength grade concrete using DMS, WMS, and M-S as fine aggregates can reach 230–260 mm. The morphological effect of sand particles in SSTS and the filling and micro-roller effects of IS can significantly improve the aggregate void ratio, reduce friction between the mortar and aggregate and the water reducer dosing, and improve the workability of the concrete mixture. Clay lumps in WMS will increase water reducer dosing and the cohesion of concrete, and treatments involving dewatering and the crushing of clay lumps in SSTS can significantly improve this shortcoming.
- Given that SSTS and IS improve the pore structure of concrete and the spatial network structure of cementing materials, the 28-d compressive strength can meet the design requirements of the C30–C60 strength grade. The compressive strength of the M-S group concrete is slightly higher than those of the DMS and WMS groups, while the flexural strength and toughness of the DMS and WMS groups are superior. Moreover, the drying shrinkage, chloride ion erosion resistance, water penetration resistance, and carbonation resistance are better than in the M-S groups of concrete. Lastly, clay lumps in the WMS group causes an early cracking resistance and frost resistance lower than those resistances in the DMS and M-S groups.

## Figures and Tables

**Table 1:** Physical properties of SSTS.

Parameters	Values	GB/T14684-2011 Requirements
Fineness modulus	1.2	/
Specific granularity	7.6	/
Apparent density (kg/m^3^)	2620	≥2500
Loose bulk density (kg/m^3^)	1470	≥1400
Dense bulk density (kg/m^3^)	1680	/
Loose porosity (%)	44	≤44
Dense porosity (%)	36	/
Clay content (%)	17.0	≤5
Clay lump content (%)	47.8	≤2
Chloride ion content (%)	0.0046	≤0.01
Organic content	qualified	/
Moisture content (%)	17.0	/

**Table 2:** Chemical components of SSTS, silt and SB.

Samples	$SiO_{2}$	$Al_{2}O_{3}$	$Na_{2}O$	$K_{2}O$	C	$Fe_{2}O_{3}$	MgO	Loss
SSTS	69.26	10.92	4.43	4.12	3.47	2.35	1.27	3.25
Silt	57.07	10.96	2.93	3.36	8.15	5.66	2.76	7.06
SB	53.27	14.03	4.86	2.21	5.42	5.27	2.97	10.48

**Table 3:** Particle gradation, fineness modulus, porosity, and clay content of each mixed sand.

Nos.	SSTS Replacement Rates/%	Accumulated Retained Percentage of Different Sieve Size/%						Fineness Modulus	Loose Porosity/%	Dense Porosity/%	Clay Content/%
		4.75	2.36	1.18	0.60	0.30	0.15				
1	0	4.5	31.2	70.9	96.9	97.2	97.4	3.8	49.3	44.3	0.3
2	20	3.6	24.9	56.9	81.2	83.5	91.5	3.3	44.2	37.1	3.5
3	40	2.7	18.7	42.9	65.4	69.8	85.7	2.7	40.4	33.3	7.0
4	60	1.8	12.5	28.9	49.7	56.1	79.9	2.2	40.4	32.8	10.5
5	80	0.9	6.3	14.9	33.9	42.4	74.0	1.7	41.3	33.8	13.4
6	100	0	0.3	1.4	7.9	41.9	70.9	1.2	43.8	36.0	17.0

Remarks: Sample No. 1 is CMS; Sample No. 6 is SSTS.

**Table 4:** Three types of fine aggregates used in concrete preparation.

Fine Aggregates	Treatments	Fineness Modulus
DMS	Dehydrated SSTS and crushed large sand cluster and clay lump to replace 40% mass of CMS	2.7
WMS	SSTS without dehydrating and crushing process to replace 40% mass of CMS	2.7
M-S	Dehydrated M-S	2.6

**Table 5:** Mix proportion of concrete.

Nos.	W/B	Weight (kg/m^3^)								Water Reducer (%)
		Cement	Fly Ash	Slag	SSTS	CMS	M-S	Gravel	Water	
C30DMS	0.47	232	77	77	330	494	0	930	182	1.56%
C30WMS	0.47	232	77	77	381	494	0	930	131	3.23%
C30M-S	0.47	232	77	77	0	0	824	930	182	2.07%
C40DMS	0.42	260	87	87	291	436	0	965	182	1.46%
C40WMS	0.42	260	87	87	336	436	0	965	137	2.88%
C40M-S	0.42	260	87	87	0	0	727	965	182	2.16%
C50DMS	0.34	300	100	100	257	385	0	1047	170	2.25%
C50WMS	0.34	300	100	100	297	385	0	1047	130	2.99%
C50M-S	0.34	300	100	100	0	0	642	1047	170	3.14%
C60DMS	0.31	317	106	106	259	388	0	1056	164	2.41%
C60WMS	0.31	317	106	106	299	388	0	1056	124	2.49%
C60M-S	0.31	317	106	106	0	0	613	1056	164	2.94%

**Table 6:** Compressive and flexural strengths of each group.

Nos.	Compressive Strength (MPa)				28 d Flexural Strength (MPa)	r
	3 d	7 d	28 d	60 d		
C30DMS	22.9	32.9	38.8	43.3	5.6	0.170
C30WMS	23	33.3	40.8	44.5	5.8	0.167
C30M-S	26.7	38.1	45.2	49.9	5.4	0.142
C40DMS	31.1	38.6	48.1	55.9	6.3	0.154
C40WMS	30.9	39.8	48.9	58.6	6.4	0.153
C40M-S	33.4	43.7	53.1	59.8	6.0	0.134
C50DMS	38.8	51.9	59.4	66.8	7.4	0.146
C50WMS	39.6	52.7	60.3	68.2	7.5	0.146
C50M-S	42.8	54.7	62.3	69.3	7.0	0.132
C60DMS	46.5	59.6	69.5	76.1	7.6	0.128
C60WMS	47.9	60.4	70.7	78.2	7.7	0.129
C60M-S	48.5	62.5	70.5	77.9	7.2	0.121

Remarks: Data are the average of the measured values of three specimens in each group and multiplied by the dimension factor of 0.95 for compressive strength and 0.85 for flexural strength. “r” is the ratio of 28-d flexural strength to 28-d compressive strength for each group of concrete.

**Table 7:** Compressive strength of 28-d-aged *t*-statistical analysis.

Hypotheses	DF	*t*	$\mathrm{Reject}\;\mathrm{Region}\;\left|t\right|\geq t_{0.975}\left(DF\right)$	Remarks
$\mu_{C30DMS}-\mu_{C30WMS}=0$	2	−1.965	4.303	insignificant
$\mu_{C30DMS}-\mu_{C30M-S}=0$	2	−7.026	4.303	significant
$\mu_{C30WMS}-\mu_{C30M-S}=0$	3	−7.706	3.182	significant
$\mu_{C40DMS}-\mu_{C40WMS}=0$	2	−0.726	4.303	insignificant
$\mu_{C40DMS}-\mu_{C40M-S}=0$	3	−3.886	3.182	significant
$\mu_{C40WMS}-\mu_{C40M-S}=0$	3	−4.689	3.182	significant
$\mu_{C50DMS}-\mu_{C50WMS}=0$	2	−0.651	4.303	insignificant
$\mu_{C50DMS}-\mu_{C50M-S}=0$	3	−2.741	3.182	insignificant
$\mu_{C50WMS}-\mu_{C50M-S}=0$	3	−1.272	3.182	insignificant
$\mu_{C60DMS}-\mu_{C60WMS}=0$	3	−0.823	3.182	insignificant
$\mu_{C60DMS}-\mu_{C60M-S}=0$	3	3.182	3.182	insignificant
$\mu_{C60WMS}-\mu_{C60M-S}=0$	3	0.128	3.182	insignificant

**Table 8:** First cracking time and initial crack width of each group.

Nos.	First Cracking Time (h)	Initial Crack Width (mm)
C30DMS	74	2.13
C30WMS	53	3.52
C30M-S	62	3.02
C40DMS	72	2.60
C40WMS	48	3.57
C40M-S	52	2.93
C50DMS	68	2.94
C50WMS	48	3.64
C50M-S	49	3.53
C60DMS	62	3.19
C60WMS	46	3.72
C60M-S	48	3.64

**Table 9:** Depth of carbonation of concrete at different ages.

Nos.	Depth of Carbonation (mm)		
	3 d	7 d	28 d
C30DMS	1.13	2.10	3.51
C30WMS	1.42	2.69	4.00
C30M-S	3.13	4.12	6.20
C40DMS	1.11	2.07	3.21
C40WMS	1.23	2.12	3.52
C40M-S	1.47	2.45	4.41
C50DMS	0.69	1.17	2.42
C50WMS	0.71	1.28	2.63
C50M-S	1.13	2.23	3.31
C60DMS	0.53	0.94	1.73
C60WMS	0.58	0.96	1.82
C60M-S	0.77	1.32	2.30

## Data Availability

Not applicable.

## Abbreviations

The abbreviation in this paper include: Slurry shield tunnel slag (SSTS); Coarse Manufactured Sand (CMS); Inert Silt (IS); Tunnel Slag (TS); Sodium Bentonite (SB); Manufactured sand (M-S); Dry Mixed Sand (DMS), Wet Mixed Sand (WMS); Polyacrylamide (PAM).
